# Analysis of peginterferon β-1a exposure and Gd-enhanced lesion or T2 lesion response in relapsing-remitting multiple sclerosis patients

**DOI:** 10.1007/s10928-016-9477-x

**Published:** 2016-06-14

**Authors:** Yaming Hang, Xiao Hu, Jie Zhang, Shifang Liu, Aaron Deykin, Ivan Nestorov

**Affiliations:** Biogen, Cambridge, MA USA

**Keywords:** Peginterferon β-1a, Multiple sclerosis, Longitudinal count data, Mixture model, Population pharmacokinetics, Exposure–response

## Abstract

The effect of subcutaneous (SC) peginterferon β-1a exposure on reduction of gadolinium-enhanced (Gd+) lesion count over time was evaluated in patients with relapsing-remitting multiple sclerosis (RRMS) in a Phase 3 study (ADVANCE). Patients were randomized to receive SC injections of placebo (n = 500), 125 mcg every-2-weeks (n = 512), or 125 mcg every-4-weeks (n = 500) for 1 year, and then active treatment in the second year. Steady state 4-week AUC (AUCss) was derived for each individual based on sparse pharmacokinetic (PK) sample and a population PK model. Several longitudinal count models, including marginal, mixed effect, and mixture models, were compared to explore the relationship between AUC_ss_ and Gd+ lesion count (or T2 lesion count). A mixture model which divided subjects into two subpopulations by low and high baseline lesion activity was found to yield best goodness-of-fit for the data. In this model, the point estimate and 95 % CI for drug effect slope on log(λ) are −0.0256 (−0.0304, −0.0216) for Gd+ lesion and −0.0147 (−0.0170, −0.0124) for T2 lesion. This suggested that reduction of Gd+ lesion (or T2 lesion) count over time is significantly related to SC peginterferon β-1a exposure, and that the increased reduction lesion count with the every-2-week regimen versus the every-4-week regimen was driven by the higher exposure achieved in that treatment arm (mean Gd+ lesion count 0.2 and 0.7 at Year 2, respectively). The every-2-week regimen produced an exposure range that was close to the plateau range of the exposure–response curve, supporting its selection as the regulatory approved dosage.

**Electronic supplementary material:**

The online version of this article (doi:10.1007/s10928-016-9477-x) contains supplementary material, which is available to authorized users.

## Introduction

Multiple sclerosis (MS) is a chronic disease of the central nervous system. It is predominantly a disease of young adults, primarily women, with disease onset typically occurring between the ages of 20 and 40. MS primarily affects myelinated fiber tracts. Histologically, it is characterized by focal areas of demyelination, astrogliosis, the relative preservation of axons, and varying degrees of inflammation [1].

Peginterferon β-1a, a PEGylated form of interferon β-1a (IFN β-1a), has been approved for the treatment of relapsing multiple sclerosis (RMS). Treatment with IFN β-1a 30 mcg intramuscular (IM) injection weekly has proven to be effective in delaying the progression of disability and in reducing the rate of clinical relapses in MS. Peginterferon β-1a has a longer half-life and greater exposure compared to IFN β-1a; therefore, with a reduced administration frequency, it potentially reduces side effects (e.g. flu-like symptoms) while increasing convenience and improving treatment compliance [2, 3].

In the ADVANCE study, a pivotal Phase 3 study, after 1 year of treatment, SC peginterferon β-1a every-2-weeks reduced annualized relapse rate (ARR; primary endpoint) by 36 %. Risk of relapse, risk of disability progression, and the number of new or newly enlarging T2 lesions (secondary endpoints), together with gadolinium-enhanced (Gd+) lesions (tertiary endpoint), were also reduced when compared with placebo. The safety profile reflected that of established IFN β-1a therapies [4]. The 2-year results showed that peginterferon β-1a efficacy was maintained beyond 1 year, with greater effects observed with every-2-week versus every-4-week dosing, and a similar safety profile to Year 1 results [5].

Gd+ lesions (also called enhancing lesions), T2-hyperintense lesions, and T1-hypointense lesions (T1 black holes) are the basis for the three classic measures of MS pathology visible by conventional magnetic resonance imaging (MRI), and are the cornerstone of MRI-based outcomes in MS clinical trials [6]. Gadolinium (Gd) chelates are widely used as contrast agents in MRIs of the brain and spinal cord. Data from animal studies and from MS brain biopsy studies have demonstrated that Gd enhancement is associated with histopathological evidence of blood–brain barrier breakdown and inflammation. Studies indicate that enhancement occurs in almost all the new lesions from patients with relapsing-remitting or secondary progressive MS. Generally speaking, the enhancement of a new lesion lasts 2–3 weeks in most cases [7, 8].

T2-hyperintense lesions (T2 lesions) provide a complementary set of measures to enhancing lesions in both clinical trials (counts or volumetrics) and in clinic (principally counts). New T2 lesion counts in most circumstances are strongly correlated with enhancing lesion counts in high-frequency serial studies. While enhancing lesions provide a measure of inflammation only around the time of the MRI, new T2 lesions represent a measure of disease activity over the interval.

The objectives of the current analyses were to explore and evaluate the relationship between the population pharmacokinetic (PK) exposure predicted for peginterferon β-1a and the observed Gd+ lesion count over time in the ADVANCE study. Similar analyses were performed for T2 lesion count, except that instead of assuming the mean count of new or newly enlarged lesions declined over time, it was assumed to be proportional to duration of observation. To avoid redundancy, this paper will focus on the method and results for the analysis of Gd+ lesion count, and the modeling outcome for T2 lesion count will be briefly mentioned.

## Methods

### Study design and patients

ADVANCE was a 2-year, Phase 3, multicenter, randomized, double-blind, parallel-group study with a 1-year, placebo-controlled period. The full methods from the ADVANCE study have been published previously [4, 5]. During Year 1 of the study, patients were randomized (1:1:1) to receive SC injections of placebo (n = 500), peginterferon β-1a at a dose of 125 mcg every-2-weeks (n = 512), or peginterferon β-1a at a dose of 125 mcg every-4-weeks (n = 500). At the end of Year 1, patients on placebo were re-randomized to either peginterferon β-1a 125 mcg every 2 or 4 weeks, while patients on peginterferon β-1a during Year 1 continued their treatment. During the first year of the study, 44, 62, and 74 subjects dropped out of the study in the placebo, every-4-weeks and every-2-weeks arms, respectively. Among the subjects who continued into the Year 2 portion of the study, 75 and 59 subjects dropped out of the study in the every-4-weeks and every-2-weeks arms, respectively. Key eligibility criteria were a diagnosis of RRMS as defined by the McDonald criteria, age 18–65 years, a score of between 0 and 5 on the expanded disability status scale (EDSS; with higher scores indicating greater disability [9]), and at least two clinically documented relapses in the previous 3 years, with at least one of these relapses having occurred within the 12 months prior to randomization. Patients who had progressive forms of MS, pre-specified laboratory abnormalities, and prior interferon treatment for MS exceeding 4 weeks or discontinuation less than 6 months prior to baseline were excluded. The protocol was approved by each site’s institutional review board and was conducted according to the International Conference on Harmonization Guidelines for Good Clinical Practice and the Declaration of Helsinki. Every patient provided written informed consent prior to study entry [4, 5].

### Pharmacodynamic (PD) measurements

MRI scans for detection of Gd+ lesions and T2 lesions were conducted at baseline, Week 24, Week 48, and Week 96. The number of Gd+ lesions was recorded for each individual at these visits. The number of new or newly enlarging T2 hyperintense lesions was recorded at Week 24, Week 48, and Week 96.

### Population PK modeling

The population PK analysis was carried out using a nonlinear mixed-effect model approach with NONMEM software (ICON plc, Dublin, Ireland, version 7.2). The final PK parameter estimates used in this analysis were based on nonparametric bootstrapping results using the final population PK model developed elsewhere [10]. One thousand data sets were generated by repeatedly sampling with replacement stratified by intensive and sparse sampling type from the Phase 3 ADVANCE study PK data set. The median values of these bootstrapped parameters were used as model population parameters, and post hoc PK parameters for all subjects were obtained by setting MAXEVAL = 0 in NONMEM.

### Modeling of longitudinal count data

Analysis of longitudinal Gd+ lesion count data is abundant in the literature. Albert et al. [11] described Gd+ lesion count data collected at monthly intervals for approximately 30 months with two types of models: a Poisson time series in which the mean changes as a function of sinusoidal trend and past observations, and a Poisson time series with the mean fluctuating according to a hidden Markov chain. Altman et al. [12] extended the hidden Markov model in [11], and also proposed a more efficient algorithm for model parameter estimation. Both [11] and [12] focused on the within-subject change of mean lesion count over time, and the models were fitted to data at individual level. Recognizing the interpatient heterogeneity in disease activity, MacKay Altman et al. [13] further extended the hidden Markov model to a mixed hidden Markov model and the model was fit to data from a group of patients simultaneously. While not analyzing lesion count data, Trocóniz et al. [14] compared Poisson, zero-inflated Poisson (ZIP), negative binomial, and zero-inflated negative binomial model for daily seizure count data, which displayed a common feature with the Gd+ lesion count data: overdispersion and Markovian properties. Velez de Mendizabal et al. [15] also compared several discrete distribution models for monthly Gd+ lesion count over 48 months in nine MS patients; these models incorporated different distribution families (Poisson, ZIP, generalized Poisson, negative binomial, and zero-inflated negative binomial) with or without Markovian element.

With this analysis, we established the relationship between the change in mean lesion count over time and the peginterferon β-1a exposure. In addition, other than exploring the models described in the literature, we extended the models used in the literature by introducing a two-population mixture model to describe the interpatient heterogeneity observed in the ADVANCE study.

### Exposure–response modeling

**Fig. 1:**
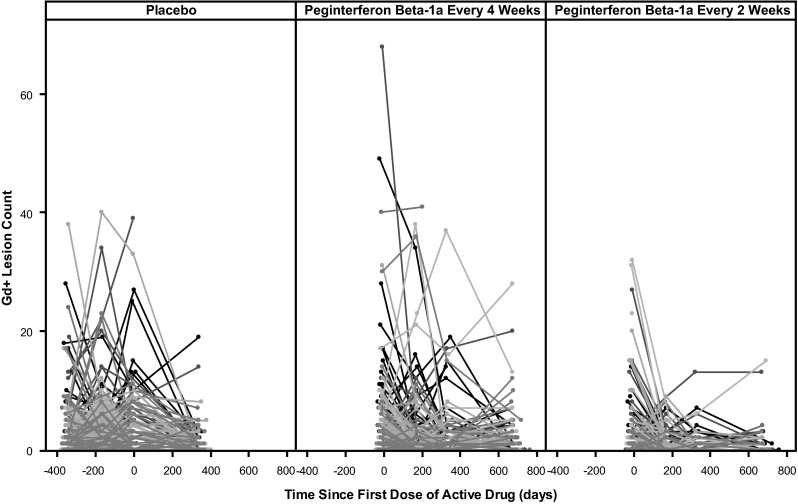
Observed Gd+ lesion count over time for each individual were overlaid and grouped by initial treatment assignment. *Each line* represents one subject. The data displayed large between-subject as well as within-subject variation. In addition, the shift of distribution of lesion count toward zero is apparent after treatment with SC peginterferon β-1a, and is more pronounced with the every-2-week arm

Spagatti plot by individual (as in Fig. 1 for Gd+ lesion count) suggested that the distribution of lesion count is similar across different visits when patients were on placebo. This was also confirmed by comparison of empirical cumulative distribution function curves and summary statistics (data shown). These analyses suggested that disease activity for both type of lesions are stable with 1 year placebo treatment; therefore, it was assumed that underlying distribution for lesion count did not change when subjects were on placebo treatment.

The half-life of peginterferon β-1a was previously reported to be ~78 h in RRMS patients with no accumulation in either the every-2-week or every-4-week treatment regimens [16]. Since change in the Gd+ lesion formation process is gradual, it was deemed unlikely that the Gd+ lesion count would change in response to change in instantaneous peginterferon β-1a concentration. A more likely indirect response model, while potentially suitable for describing the underlying relationship, was not appropriate given the limited number of lesion count observations available per individual. As a result, we estimated individual AUC_ss_ as the exposure parameter and the temporal pattern for drug effect onset was described by an empirical first-order exponential function as suggested by the data. The AUC_ss_ was derived according to Eq. (1) where *DOSE* equals 125 mcg, *N* represents the total number of doses received over 4 weeks (N = 2 for subjects in the every-2-week group; N = 1 for subjects in the every-4-week group), and *CL* represents post hoc clearance estimate for each subject:

1 $$AUC_{ss} = \frac{DOSE \times N}{CL}$$

We also explored Poisson distribution and negative binomial distribution for our response data.

In order to account for the excess zeros observed in the data set, the ZIP distribution was also tested. The ZIP distribution is defined as in Eq. (2):

2 $${\text{P}}\left( {{\text{x}} = {\text{k}}} \right) = \left\{ {\begin{array}{*{20}c} {{\text{P}}_{0} + \left( {1 - {\text{P}}_{0} } \right) \times \exp \left( { -{\uplambda }} \right),\quad {\text{if}}\,\,k = 0} \\ {\left( {1 - {\text{P}}_{0} } \right) \times \frac{{{{\uplambda }}^{\text{k}} }}{{{\text{k}}!}} \times \exp \left( { - {{\uplambda }}} \right), \quad {\text{if}}\,\,k > 0} \\ \end{array} } \right.$$

where x is the random variable for lesion count, k is an observed outcome, *P*_0_ is the inflated proportion of zero counts, and *λ* is the mean parameter. This model assumes that the observations of lesion count are coming from two processes: one process for the excess zeros and the other process that behaves as a regular Poisson. The Negative Binomial distribution allows a flexible relationship between mean and variance. Its probability mass function is given in Eq. (3):

3 $${\text{P}}\left( {{\text{x}} = {\text{k}}} \right) = \frac{{\Gamma \left( {{\text{k}} + \frac{1}{\text{OVDP}}} \right)}}{{\Gamma \left( {{\text{k}} + 1} \right) \times \Gamma \left( {\frac{1}{\text{OVDP}}} \right)}} \times \left( {\frac{1}{{1 + {\text{OVDP}}*{{\uplambda }}}}} \right)^{{\frac{1}{\text{OVDP}}}} \times \left( {\frac{{{\uplambda }}}{{{{\uplambda }} + \frac{1}{\text{OVDP}}}}} \right)^{\text{k}} , \quad {\text{k}} \ge 0$$

where x is the random variable for lesion count, k is an observed outcome, *λ* is the mean parameter, and OVDP is a parameter reflecting over-dispersion. Similarly, the Negative Binomial distribution can be modified to accommodate an excess proportion of zeros.

E_max_ and log-linear function forms were explored to describe the effect of AUC_ss_ on Gd+ lesion count:

4 $${{\uplambda }}_{\text{ij}} = {{\uplambda }}_{{{\text{i}}0}} \times \left( {1 - {\text{E}}_{ \hbox{max} } \times \frac{{{\text{AUC}}_{\text{ij}} }}{{{\text{EC}}_{50} + {\text{AUC}}_{\text{ij}} }}} \right)$$

5 $${{\uplambda }}_{\text{ij}} = {{\uplambda }}_{{{\text{i}}0}} \times \exp \left( {{{\upbeta }} \times {\text{AUC}}_{\text{ij}} } \right)$$

In Eqs. (4) and (5), λ_ij_ is the mean parameter of distribution for subject i at time j (days since first dose of active dose), λ_i0_ is the baseline λ, AUC_ij_ is the AUC_ss_ value for subject i at time j (it is either 0 for subjects on placebo treatment or at baseline, or the estimated steady state level, if at time j the subject was on peginterferon β-1a treatment). In the E_max_ model, parameter *E*_max_ represents the maximal proportional change in baseline λ, and *EC*_50_ stands for the *AUC*_*ss*_ at which 50 % of the maximum reduction can be achieved. In the log-linear model, parameter β is the slope of AUC_ss_ effect in log scale.

In the case of a zero-inflated distribution, the drug effect can not only influence parameters λ, but also parameter *P*_0_, where the relationship can be expressed as:

6 $$\log \left( {\frac{{P_{0,ij} }}{{1 - P_{0,ij} }}} \right) = P_{0} + \gamma \times AUC_{ij} ,$$

In Eq. (6), *P*_0,*ij*_ stands for the inflated proportion of 0 lesion count at time j for subject i, *P*_0_ is the inflated proportion of 0 count at baseline or when receiving placebo treatment, and γ is the slope parameter for drug effect.

Marginal (naïve pooled) model and mixed-effect model were tested initially. With the mixed-effect model, random effect on baseline λ_i0_ was assumed to follow a log-normal distribution. Given the observed large proportion of subjects with zero lesion count during the entire study, a mixture model was further tested. With the mixture model, random effect on baseline λ_i0_ was assumed to come from two sub-populations: a sub-population with low lesion activity and a sub-population with relatively higher lesion activity (Eq. (7)). Two different over-dispersion parameters, OVDP1 and OVDP2, were used for the two subgroups. Since each individual only contributed one AUC_ss_ value, random effects on slope parameter were considered non-identifiable and therefore were not included.

7 $$\lambda_{i0} = \lambda_{i0,1} \times I\left\{ {Y = 1} \right\} + \lambda_{i0,2} \times I\left\{ {Y = 0} \right\}$$

where $$Y\sim Bernoulli(1, \theta )$$, $$\lambda_{i0,1} \sim Log Normal(\mu_{1} ,\omega_{1}^{2} )$$, $$\lambda_{i0,2} \sim Log Normal(\mu_{2} ,\omega_{2}^{2} )$$, and *μ*_2_ = *R* × *μ*_1_.

Preliminary visual inspection suggested peginterferon β-1a treatment gradually decreases Gd+ lesion count over time and the maximum effect was observed at Week 54 or beyond; therefore, a first-order exponential function was adopted to describe the time course of drug effect onset, which is described in Eq. (8) for the log-linear model case. In Eq. (8), *t*_*ij*_ is the day after first dose of active dose (would be 0 at baseline or for placebo treatment) and *t*_1/2_ is the pharmacologic half-life effect parameter to be estimated. The term is a multiplicative factor added on the exposure effect; therefore, as *t*_*ij*_ increases, the value of this term goes to 1 and maximum drug effect (or steady state effect) is achieved.

8 $${{\uplambda }}_{\text{ij}} = {{\uplambda }}_{{{\text{i}}0}} \times \exp \left( {\upbeta \times {\text{AUC}}_{\text{ij}} \times \left[ {1 - \exp \left( { - \frac{0.69}{{t_{{\frac{1}{2}}} }} \times t_{ij} } \right)} \right]} \right)$$

The same models were applied to the longitudinal new or newly enlarged T2 lesion count data, except that instead of assuming a first-order exponential decline for the onset of drug effect, the mean lesion count observed in each time interval is assumed to be proportional to the length of the observation interval.

### Model evaluation

To evaluate the goodness-of-fit for the model, the marginal proportion of each observed outcome is compared to either population-predicted marginal probabilities or the average of individual predicted probabilities.

An alternative approach of model evaluation was a simulation-based visual predictive check (VPC). Five hundred data sets were simulated using the final model and corresponding parameter estimates. The data were first binned according to the value of AUC_ss_. Data were divided into 21 subgroups: one subgroup for zero AUC and another 20 subgroups for positive AUC such that an approximately equal number of subjects are in each subgroup. Within each bin, for any pre-specified grouping of outcomes (e.g. lesion count = 0, lesion count = 1, 2 ≤ lesion count ≤ 4, and lesion count > 4), the observed marginal proportion was overlaid with the corresponding 90 % prediction interval based on simulation. In addition, to evaluate whether the variability described by the model reflects the variability in observed data, the mean and variance of lesion count in each bin in each simulated data set were obtained and compared to that of the observed data.

Finally, a non-parametric bootstrap was used to evaluate the uncertainty of the model parameter estimation [17]. One thousand bootstrap data sets were generated; each contained the same number of subjects as the original data set and they were randomly drawn with replacement. For each of the 1000 bootstrap data sets, the model parameters were estimated. The mean and the 95 % confidence interval (CI) for all the parameters based on the bootstrap replicates were compared to the estimates from the original data set.

### Statistical analysis software

Data sets for Gd+ lesion count were assembled using SAS (SAS Institute Inc., Cary, NC, USA, version 9.3). Both estimation of individual AUC_ss_ and the PK/PD analysis were carried out in NONMEM (ICON plc, Dublin, Ireland, version 7.2). Laplacian approximation method was used for parameter estimation. VPC and bootstrap procedure were implemented with PsN version 4.4.0. [18, 19]) and plotted with Xpose 4.5.3 [20, 21]. Data plotting was implemented with R software (R Foundation for Statistical Computing, Vienna, Austria, version 3.1.1).

## Results

### Patient demographics

A full description of participant flow in the ADVANCE study, and details of baseline demographic and clinical characteristics in each treatment group, have been published previously [4]. The population enrolled in this study was consistent with a general multiple sclerosis population.

A total of 1505 subjects were included in this analysis (497, 510, and 498 subjects enrolled to placebo, peginterferon β-1a 125 mcg every-2-weeks, and peginterferon β-1a 125 mcg every-4-weeks at baseline, respectively).

### Gd+ lesion count

At Year 2, relative to the peginterferon β-1a every-4-week group, the number of Gd+ lesions was reduced by 71 % (p < 0.0001) in the peginterferon β-1a every-2-weeks group (0.2 and 0.7 for the every-2-week and every-4-week respectively). Individual Gd+ lesion counts across visits are displayed in Fig. 1 by treatment assignment for the 2 year period. The data revealed large between-subject as well as within-subject variation. In addition, the shift in the distribution of lesion count toward zero is apparent after treatment with peginterferon β-1a, and it is more pronounced within the every-2-week arm.

Another characteristic of this data set is the high proportion of zero count. Across the three treatment arms, 38, 57, and 43 % of subjects had no Gd+ lesions over all four MRI scans in the placebo, the every-2-week, and the every-4-week arms, respectively. At the same time, there was a small proportion of lesion counts that were ≥30 in each arm. This indicated that the distribution of lesion counts is heavily skewed toward zero; however, there is also a heavy tail toward the large counts. As a result, high over-dispersion is a feature of the data (in Supplementary Fig. S2, marginal mean of Gd+ lesion count was plotted against the variance of Gd+ lesion count for subgroups defined by AUCss, which suggested high over-dispersion).

### Model comparison

A comparison of the different models tested in the analysis of Gd+ lesion counts is summarized in Table 1.

**Table 1:** Comparison of different models tested for Gd+ lesion count

Model	Description	OFV	Estimated slope of AUC effect
Model 1	Naïve pooled poisson model	23,223.8	−0.0254
Model 2	Naïve pooled zero-inflated Poisson model, log-linear model for AUC effect on λ, and logistic model for AUC effect on P_0_	16,840.5	−0.0116 on λ 0.0163 on P_0_
Model 3	Naïve pooled negative binomial model, time effect was removed to allow convergence	11,960.8	−0.0209
Model 4	Mixed effect negative binomial model, random effect on baseline rate parameter *λ*_*i*0_ (assuming log-normal distribution), over-dispersion parameter OVDP assumed constant	11,310.1	−0.0267
Model 5	Mixture model on baseline rate parameter *λ*_*i*0_ with two log-normally distributed sub-populations, OVDP assumed to be different for each sub-population	10,971.4	−0.0256

In all models, the AUC effect on mean Gd+ lesion count was assumed to follow a log-linear model, and drug-effect onset was described by a first-order exponential function

With the marginal Poisson model (Model 1, Table 1), the minimum objective function value (OFV) was 23,223.8 and the estimated slope parameter for exposure effect was −0.0254. As shown in Fig. 3, this model did a poor job predicting the probability of either small counts (0, 1, 2, and 3) or large counts (≥10).

The marginal ZIP model (Model 2, Table 1) was then fitted to the data, where a parameter to reflect extra proportion of zeros was added to a regular Poisson distribution (parameter *P*_0_ in Eq. (2)). The OFV for this model was 16,840.5, which corresponded to a reduction of 6383.3 points compared to the marginal Poisson model. The parameter *P*_0_ is estimated to be 0.596. As the effect of peginterferon β-1a exposure is parameterized to both reduce the mean of regular Poisson component (parameter λ in Eq. (2)) as well as increase the extra proportion of zero (parameter P_0_ in Eq. (2)), two drug effect parameters were required and their estimates were −0.0116 and 0.0163, respectively.

The marginal negative binomial model (Model 3, Table 1) yielded an OFV of 11,960.8, which was a further reduction of 4879.7 from the marginal ZIP model. The estimated drug effect slope was −0.0209. The estimated OVDP parameter was 13.4, indicating the variability of data is much larger than the mean of data. Figure 3a, b suggested a much improved fit in both ends of the distribution curve, although slight over-estimation for the probability of zero count was apparent. However, the marginal zero-inflated negative binomial model appeared to be over-parameterized, and it failed to converge.

The marginal models assume that all the subjects share a common baseline level of λ. However, in-depth review of the individual profiles revealed that, although within-subject variability across different visits could be large, in general, subjects with more active disease tend to have more lesions observed in multiple visits, or in the contrast, consistently low lesion counts were observed across multiple visits. This is an indication that different subjects have different inherent disease activity levels. Therefore, adding a random effect into the model to account for between-subject variability in baseline disease activity would be a natural next step to explore. As it was apparent from prior tested models that negative binomial models are more suitable for such over-dispersed data, only negative binomial models were further evaluated.

**Fig. 2:**
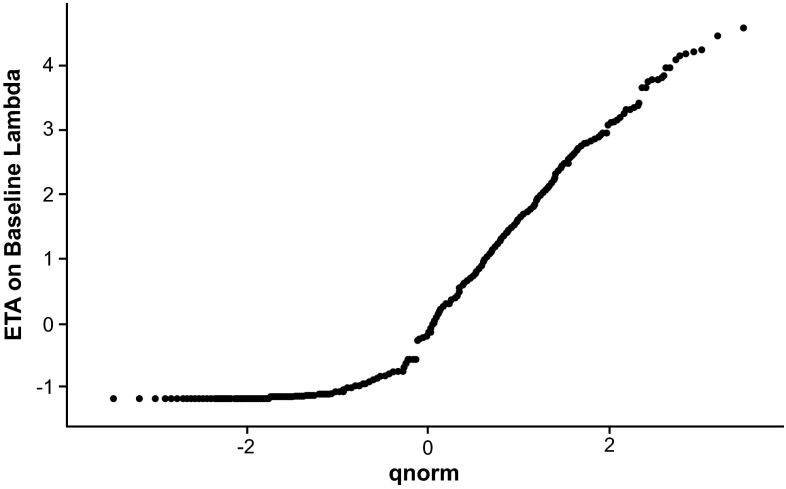
Quantile–quantile plot for empirical Bayes estimate of *η*_*λ*0_ in the mixed effect negative binomial model (Model 4). The random effect on baseline λ was assumed to follow a log-normal distribution; therefore, *η*_*λ*0_ should follow a normal distribution. However, the lower end of the distribution significantly deviated from the Normal distribution. This part of the distribution comprised predominantly subjects with no lesions observed during the trial and their η estimate would be different only when the corresponding AUC_ss_ is different. This graph indicates that the individual level λ cannot be differentiated for these subjects; therefore, grouping them into a sub-population was performed

In the mixed effect negative binomial model (Model 4, Table 1), the individual baseline mean count *λ*_*i*0_, which reflects underlying Gd+ lesion disease activity prior to treatment with peginterferon β-1a, was assumed to follow a log-normal distribution. The model also converged successfully and all parameters were estimated with relative standard error (RSE) less than 19 %. The OFV for this model was 11,310.1, reflecting a reduction of 650.7 from the marginal negative binomial model. The estimated drug effect slope was −0.0267. The omega parameter (variance for *η*_*λ*_) was estimated to be 1.712, which translated into a coefficient of variation of 131 % in the between-subject variation in baseline disease activity. Interestingly, the OVDP parameter estimation was reduced to 0.788; this suggests that, once the large between-subject variation in the baseline disease activity was accounted for, the variability of observations at individual level was much reduced. This is in contrast to the large value of estimated OVDP parameter in the marginal negative model, where it groups together both the between- and within-subject variation. A further evaluation of the distribution of Empirical Bayesian Estimates of *η*_*λ*_ indicates that its distribution is highly skewed and substantially deviated from normal distribution (Fig. 2); many subjects had small yet almost identical values of estimated *η*_*λ*_. This indicates that the log-normal distribution is not appropriate for the random effect of baseline mean Gd+ lesion count.

**Fig. 3:**
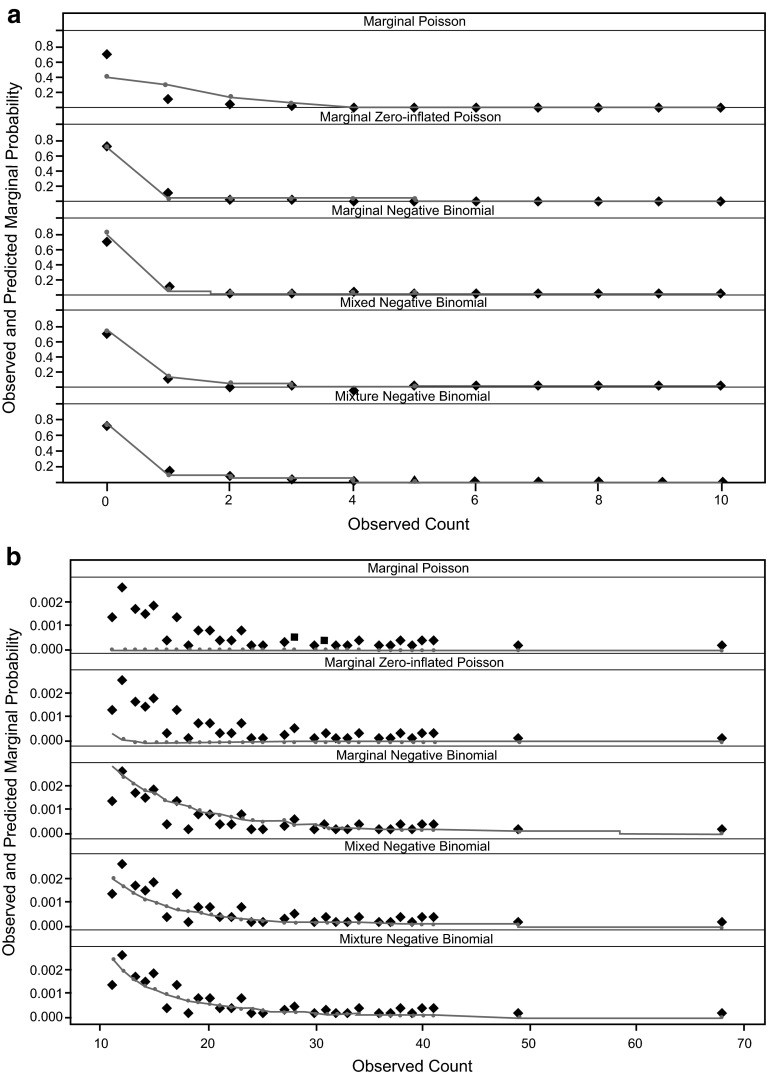
Goodness-of-fit for marginal probability of observed count in the entire data set across different models. **a** count ≤10; **b** count >10. Observed marginal probabilities (diamonds) were overlaid with model-predicted marginal probabilities (lines) for different models. For each unique lesion count observed in the data set, the marginal probability is defined as the ratio between the total number of the count and the total number of observations in the data set. To calculate the model-predicted marginal probability for a particular count, the probability of observing this count at each visit for each individual is first obtained based on individual empirical Bayes estimates of parameters and then averaged

The final model was a mixture negative binomial model (Model 5, Table 1) with two subpopulations for the between-subject variation on baseline mean parameter λ_i0_, which reflects that 40–60 % of the subjects had no recorded Gd+ lesions across all four MRI scans. Again, the model converged successfully and the OFV was 10,971.4. Although visually the improvement on the marginal probability in Fig. 3a, b is quite subtle, there appears to be better fitting, especially in the tail part of the distribution curve, with the mixture negative binomial model. The parameter estimates, together with the RSEs, are given in Table 2. The median as well as the 95 % CI based on the bootstrap for each parameter are listed in Table 2, confirming that all parameters were estimated with good precision.

**Table 2:** Non-parametric bootstrap analysis and parameter estimates of the final model for Gd+ lesion count (Model 5 mixture negative binomial model)

Model parameter	Description	Point estimate	Non-parametric bootstrap (500 replicates)	
			Median	95 % CI
*μ*_1_	Baseline mean Gd+ lesion count for a typical subject in the lower baseline lesion activity sub-population	0.48	0.474	(0.382, 0.609)
R	Ratio of the mean Gd+ lesion count for a typical subject in higher baseline lesion activity group to lower baseline lesion activity group	3.53	3.58	(2.58, 4.73)
*μ*_2_	Baseline mean Gd+ lesion count for a typical subject in the higher baseline lesion activity sub-population	1.69	1.70	
r1	Dispersion parameter for baseline λ in the lower baseline lesion activity sub-population	44.8	44.7	(39.6, 50.8)
r2	Dispersion parameter for baseline λ in the higher baseline lesion activity sub-population	0.499	0.496	(0.398, 0.586)
θ	Proportion of subjects with lower baseline lesion activity	0.602	0.602	(0.561, 0.651)
β	Slope of AUC effect on log(λ)	−0.0256	−0.0257	(−0.0304, −0.0216)
t_1/2_	Half-life of drug effect onset time (days)	115	113.8	(73.8, 179.6)
σ^2^	Variance of random effect on baseline λ in log scale for the higher baseline lesion activity sub-population	1.25	1.22	(1.00, 1.46)

The model suggests that approximately 60 % of the subjects fall into the lower baseline lesion activity sub-population (see also Supplementary Fig. S1). The typical values of λ_i0_ are estimated to be 0.48 and 1.69 for the lower baseline lesion activity and higher baseline lesion activity sub-populations, respectively. This was a sizeable separation between these two groups. The model estimated the between-subject variability on λ_i0_ for the lower baseline lesion activity group to be very small; therefore, it was set to zero. The between-subject random effect on λ_i0_ for the higher lesion activity sub-population was estimated to have a 112 % coefficient of variation, which implied large heterogeneity in disease activity among patients. Different OVDP parameters were needed for the two sub-populations and they were estimated to be 44.8 and 0.5 for the low lesion activity and high lesion activity groups, respectively. At first inspection, this appeared to be counter-intuitive. However, since the OVDP value for the lower lesion activity group encompasses both between- and within-subject variation and mean lesion count was smaller in this group, the ratio between variance and mean was indeed large.

The estimated slope of AUC_ss_ effect on log(λ) is −0.0256; this implied that, with each additional increase of 27 ng/mL h in AUC_ss_, λ was reduced by an additional 50 %. Across the every-2-week AUC_ss_ range, the range of decline was much narrower than that across the every-4-week AUC_ss_ range; in addition, the mean decline for the every-2-week group was greater than that of the every-4-week group.

**Fig. 4:**
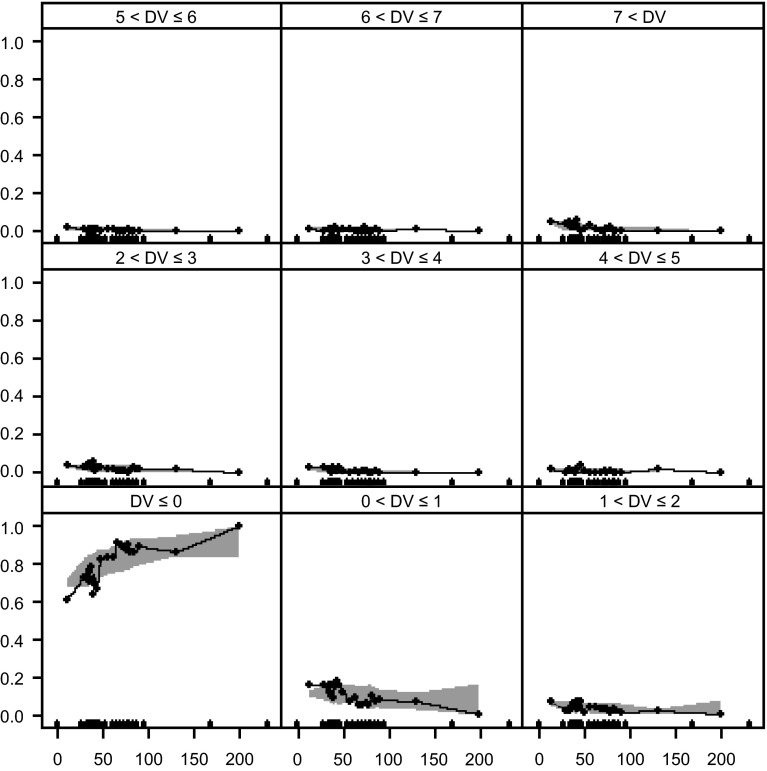
Visual predictive check for marginal probability of different lesion count categories (0, 1, 2, 3, 4, 5, 6, 7, and >7 counts) based on 1000 simulations with Model 5 the mixture negative binomial model. The Gd+ lesion count observations were divided into 21 groups according to associated AUC_ss_ (one group for zero AUC_ss_ and 20 groups for all positive AUC_ss_). The dots connected by a line were the observed proportion for different lesion count categories and the shaded region were the corresponding 90 % CI based on simulation. The dots along the x-axis are the boundary value of each AUC bin

Figure 4 displays the observed proportion of different lesion counts (0, 1, 2, 3, 4, 5, 6, 7, and >7 counts) in each AUC_ss_ bin, with the corresponding 95 % CI based on 1000 simulation runs. The clear trend of increased proportion of zero count with increasing AUC_ss_, or decreased proportion of one or more lesion counts with increasing AUC_ss_, generally lies inside the confidence band. This is also true for large count numbers and the observed proportions fall well within the 95 % CI. In addition, the observed variance and mean for each subgroup defined by AUC_ss_ were similar to those from 20 simulated data sets (see also Supplementary Fig. S3). The greater-than-proportional increase in the variance relative to mean in the observed data was well reflected in the simulated data sets. This implies that the model not only captured the central tendency of the data well, but that it also represented the variability of the data well.

**Fig. 5:**
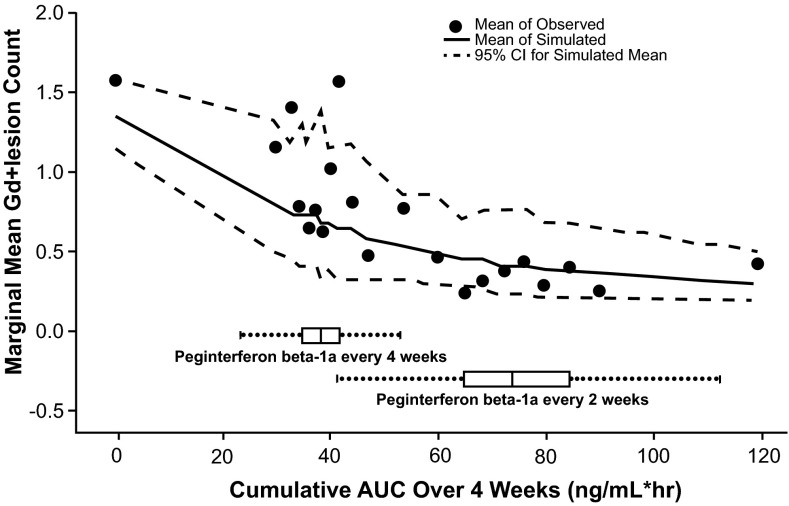
Observed marginal mean Gd+ lesion count by AUC_ss_ subgroup, overlaid with mean and 95 % CI based on 1000 simulations. Boxplot of the population PK model estimated AUC_ss_ in the every-2-week and every-4-week arms. The simulation-based mean curve is aligned with the central tendency of the observed data. In addition, the observed mean lies within the simulation-based 95 % CI. Across the range of AUC_ss_ resulting from the every-2-week regimen, marginal mean Gd+ lesion count had little change. However, in the range of AUC_ss_ resulting from the every-4-week regimen, a substantial difference in marginal mean Gd+ lesion count was either observed in the data or predicted by the model

In Fig. 5, observed marginal mean Gd+ lesion counts by AUC_ss_ subgroup were overlaid with mean and 95 % CI based on 1000 simulations. Boxplots for the population-PK-model-estimated AUC_ss_ in every-2-week and every-4-week arms are also shown. The simulation-based mean curve is aligned with the central tendency of the observed data. In addition, the observed mean lesion counts lie within the simulation-based 95 % CI. Similar marginal mean lesion count was observed across the range of AUC_ss_ resulting from the every-2-week regimen. However, in the range of AUC_ss_ resulting from the every-4-week regimen, a substantial difference in marginal mean lesion count was either observed in the data or predicted by the model among the low AUC subgroup and high AUC subgroup.

### T2 lesion count

**Fig. 6:**
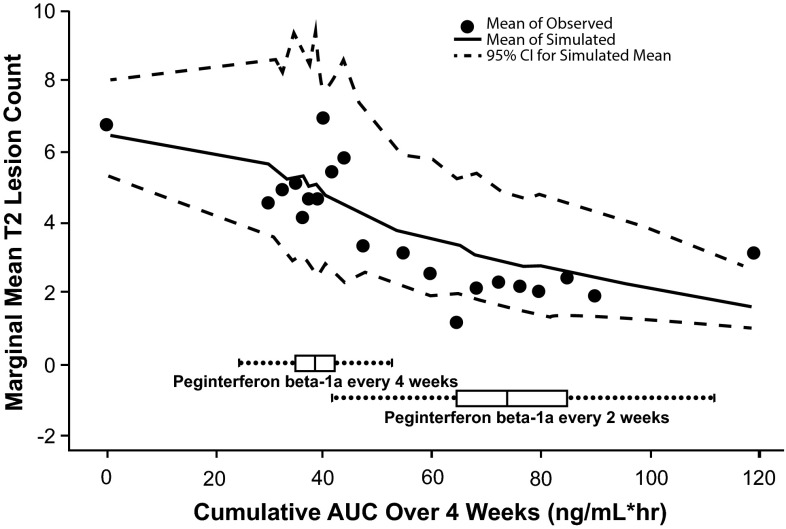
Observed marginal mean new or newly enlarged T2 lesion count by AUC_ss_ subgroup, overlaid with mean and 95 % CI based on 1000 simulations. Boxplot of the population PK model estimated AUC_ss_ in the every-2-week and every-4-week arms. The simulation-based mean curve is aligned with the central tendency of the observed data. In addition, the observed mean lies within the simulation-based 95 % CI. Across the range of AUC_ss_ resulting from the every-2-week regimen, marginal mean T2 lesion count had little change. However, in the range of AUC_ss_ resulting from the every-4-week regimen, a substantial difference in marginal mean T2 lesion count was either observed in the data or predicted by the model

Similar models were tested on the longitudinal T2 lesion count data, and the relative performance of these models in terms of goodness-of-fit are in the same order as for Gd+ lesion count data (model comparison was presented in Table 3; estimates of parameters in the final model presented in Table 4). The final model selected to describe T2 lesion count data was also a mixture negative binomial model with two sub-populations for the between-subject variation on baseline mean parameter λ_i0_. The effect of AUC_ss_ on log(λ) is estimated to be −0.0147, with a standard error (SE) of 0.0011. This suggested that AUC_ss_ is a highly significant covariate on T2 lesion count and increased AUC_ss_ led to a greater reduction of T2 lesion count. Figure 6 suggests that the model fits the data well and a smaller difference in T2 lesion count was observed among subjects receiving the every-2-week regimen compared to the every-4-week regimen.

**Table 3:** Comparison of different models tested for new or newly enlarged T2 lesion count

Model	Description	OFV	Estimated slope of AUC effect
Model 1	Naïve pooled poisson model	45,024.1	−0.017
Model 2	Naïve pooled zero-inflated Poisson model, log-linear model for AUC effect on λ, and logistic model for AUC effect on P_0_	33,561.6	−0.0117 on λ 0.011 on P_0_
Model 3	Naïve pooled negative binomial model	18,267.5	−0.0143
Model 4	Mixed effect negative binomial model, random effect on baseline rate parameter $\varvec{\lambda}_{{\varvec{i}0}}$ (assuming log-normal distribution), over-dispersion parameter OVDP assumed constant	17,021.6	−0.0157
Model 5	Mixture model on baseline rate parameter $\varvec{\lambda}_{{\varvec{i}0}}$ with two log-normally distributed sub-populations, OVDP assumed to be different for each sub-population	16,726.9	−0.0147

In all models, the AUC effect on mean T2 lesion count was assumed to follow a log-linear model, and mean parameter λ is assumed to be proportional to the duration of observation for which new or newly enlarged T2 lesion were recorded

**Table 4:** Non-parametric bootstrap analysis and parameter estimates of the final model for new or newly enlarged T2 lesion count (Model 5 mixture negative binomial model)

Model parameter	Description	Point estimate	Non-parametric bootstrap (500 replicates)	
			Median	95 % CI
*μ*_1_	Baseline mean T2 lesion count for a typical subject in the lower baseline lesion activity sub-population	0.0066	0.0065	(0.0049, 0.0089)
R	Ratio of the mean T2 lesion count for a typical subject in higher baseline lesion activity group to lower baseline lesion activity group	4.71	4.81	(3.48, 6.25)
*μ*_2_	Baseline mean T2 lesion count for a typical subject in the higher baseline lesion activity sub-population	0.031	0.031	
r1	Dispersion parameter for baseline λ in the lower baseline lesion activity sub-population	35.7	35.5	(30.6, 40.6)
r2	Dispersion parameter for baseline λ in the higher baseline lesion activity sub-population	0.459	0.454	(0.396, 0.518)
θ	Proportion of subjects with lower baseline lesion activity	0.354	0.353	(0.315, 0.394)
β	Slope of AUC effect on log(λ)	−0.0147	−0.0147	(−0.0170, −0.0124)
σ^2^	Variance of random effect on baseline λ in log scale for the higher baseline lesion activity sub-population	1.21	1.22	(1.05, 1.39)

## Discussion

Since Gd+ lesions represent acute blood–brain barrier disruption, the number of Gd+ lesions is highly unpredictable at different time points within a subject. There was one subject in the placebo arm who had 6 Gd+ lesions at the baseline visit but 40 and 33 lesions at Week 24 and Week 48, respectively; lesion count reduced to zero after switching to the every-2-weeks regimen for 1 year. Nevertheless, subjects with very few lesions at baseline tend to have a small number of lesions across all scans, while subjects with a relatively greater number of lesions at baseline are more likely to record larger lesion counts. This indicates that both between- and within-subject variation in counts are high and an appropriate model should be able to reflect these features simultaneously.

Multiple models were tested in this study, including marginal models with regular Poisson, zero-inflated Poisson, regular negative binomial, zero-inflated negative binomial, mixed effect negative binomial, and mixture negative binomial. Not surprisingly, none of the marginal models can adequately reflect such an irregular distribution of count data. Both the mixed effect negative binomial model and the mixture negative binomial model with two sub-populations performed significantly better than the marginal models. Judging from multiple factors, including the precision in parameter estimation, the value of the estimated parameter, goodness-of-fit for either marginal probability of each observed count or variance versus mean plot by subgroups of AUC_ss_, or VPC on the proportion of different counts binned by AUC_ss_, the mixture negative binomial model appeared to be the most robust model.

Compared to the paper by Velez de Mendizabal et al., the Gd+ lesion count data displayed similar features, which are high between-subject and within-subject variability, over-dispersion, and large proportion of zero count. Their data were monthly observations from nine individuals, and the data set we analysed here are much more sparse at an individual level but have a larger sample size. While incorporating a Markovian feature in the model would not be supported by the data due to long interval between MRI acquisitions, the lesion count data nevertheless indicated correlation within individuals and large sample size allowed us to divide the subjects into two sub-populations with different levels of lesion activity. Both analyses found negative binomial model fits the data better than Poisson model (even with a zero-inflated component). For the selected model in their paper, which is a negative binomial model with first and second order Markov factors, the estimated underlying mean lesion count was 0.94. Interestingly, this number is very similar to the weighted mean baseline lesion count for the two sub-populations (0.96) observed in our analysis, which suggests that reduced MRI acquisition frequency did not undermine the estimation of underlying lesion activity. This suggests that for a large-scale clinical study, the increased cost associated with more frequent MRI scans does not provide much additional value in terms of estimating the treatment effect. The estimated between-subject variability on λ was 66 % in the de Velez de Mendizabal et al. paper, and the estimate was 112 % for the high disease activity sub-group in our analysis, but it is foreseeable that for the entire population, it would be smaller and closer to their estimate.

The large heterogeneity in lesion activity among the MS patients enrolled in clinical studies is very common; similar phenomenon have been observed in clinical studies for several other MS treatments (data not shown). Apparently, it is difficult to show treatment effect in subjects with low lesion activity; therefore, for future clinical studies in MS patients where Gd+ or T2 lesion count serves as a primary endpoint (e.g. Phase 2 dose-ranging studies), it is a plausible idea to enrich patient population by selecting subjects with at least one lesion at baseline in order to improve the sensitivity of a study to detect drug effect.

## Conclusions

We tested multiple models to describe the relationship between longitudinally collected Gd+ (and T2) lesion count and steady state peginterferon β-1a AUC. A mixture negative binomial model with two sub-populations adequately captured the important features of the data, including an excessive proportion of zero counts, over dispersion, proportion of large counts greater than that implied by the typical count models, and heterogeneity in within-subject variation. This analysis suggested that SC peginterferon β-1a exposure is significantly related to the reduction in Gd+ (and T2) lesion count over time, and that the increased reduction in Gd+ and new or newly enlarged T2 lesion count with the every-2-week regimen compared with the every-4-week regimen was related to the higher exposure. The every-2-week regimen produced an exposure range that is close to the plateau range of the exposure–response curve, while the every-4-week regimen could lead to a substantial proportion of subjects with suboptimal exposure to SC peginterferon β-1a. Since the safety profiles are similar among the two regimens, and the every-2-week regimen provided superior efficacy, this regimen was the one proposed to and recommended by the FDA and EMA labels [22, 23].

## Electronic supplementary material

Below is the link to the electronic supplementary material.

Supplementary material 1 (PDF 3727 kb)
