# Supplementary material for: Analysis of peginterferon β-1a exposure and Gd-enhanced lesion or T2 lesion response in relapsing-remitting multiple sclerosis patients
Source: J Pharmacokinet Pharmacodyn. 2016 Jun 14;43:371–83. doi: 10.1007/s10928-016-9477-x (PMC4954841; doi:10.1007/s10928-016-9477-x)
Supplement: Supplementary file 1 — Supplementary material 1 (PDF 3727 kb) [file 10928_2016_9477_MOESM1_ESM.pdf]

Fig. S1 Relative frequency of total Gd+ lesion (across entire study)

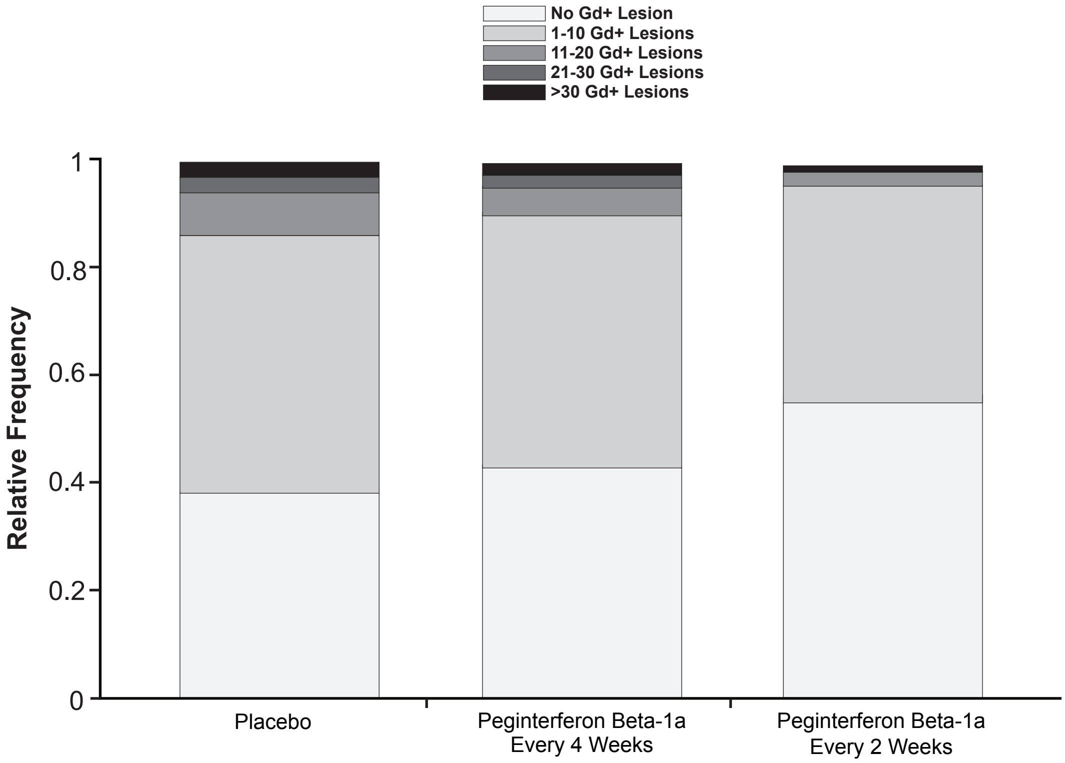

In the three arms, 38%, 57%, and 43% of subjects had no Gd+ lesions across all four MRI scans in the placebo, every-2-week, and every-4-week arms, respectively. This indicated that the distribution of lesion counts is heavily skewed toward zero, and yet at the same time has a heavy tail toward the large counts.

Fig. S2 Plot of marginal variance of Gd+ lesion count vs marginal mean

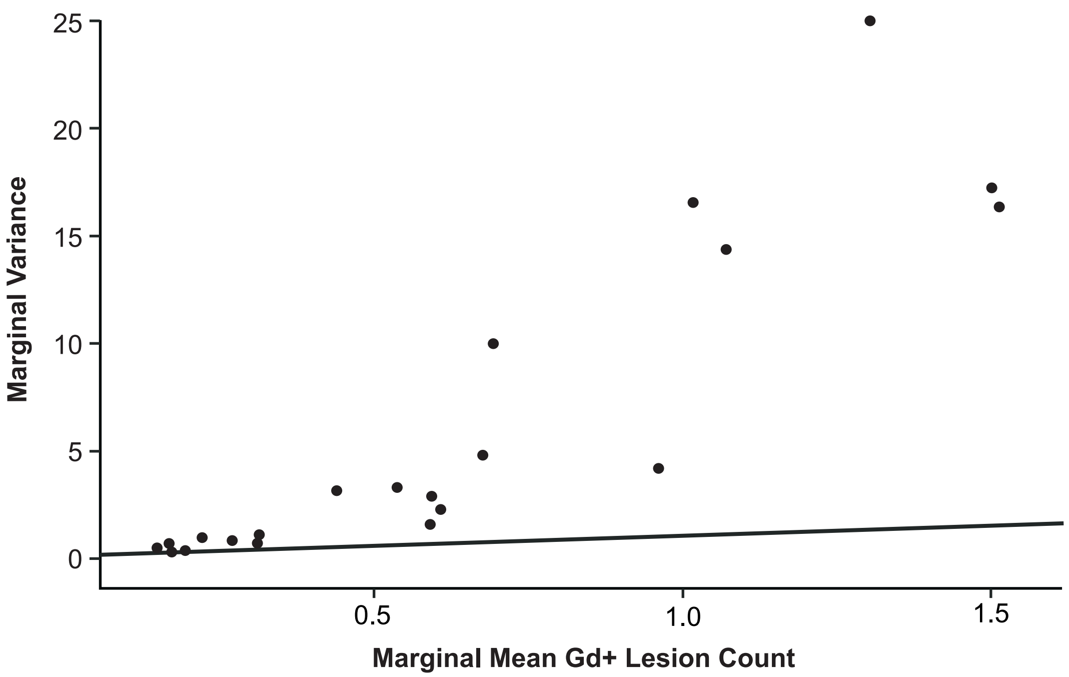

All Gd+ lesion count observations were divided into 21 groups according to associated AUCss value (observations at baseline or while on placebo treatment had a zero AUCss value). The line is the unit line. The marginal variance is much greater than the marginal mean, suggesting a substantial degree of over-dispersion in the data.

Fig. S3 Goodness-of-fit for marginal variance and mean for subgroups with different AUCss range

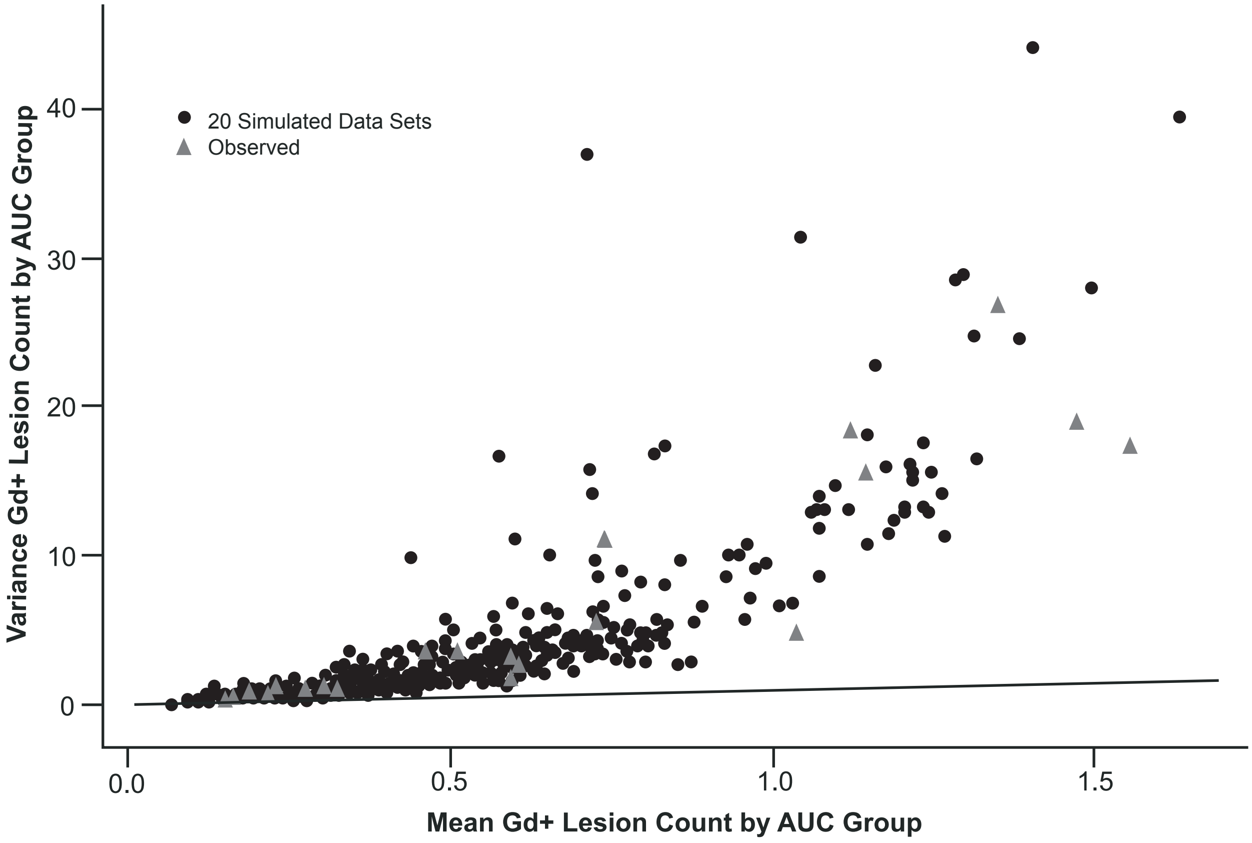

The Gd+ lesion count observations were divided into 21 groups according to associated AUCss (one group for zero AUCss and 20 groups for all positive AUCss). In each group, the mean and variance were calculated. The triangles represent observed data. The dots represent outcomes from 20 simulated data sets and the line is the unit line.
